# Integrating rapid response systems and patient centered goals of care discussions

**DOI:** 10.3389/fmed.2026.1862888

**Published:** 2026-05-29

**Authors:** Connor M. Snarskis, Kelsei P. Keene, Christopher G. Hughes

**Affiliations:** Division of Critical Care Medicine, Department of Anesthesiology, Vanderbilt University Medical Center, Nashville, TN, United States

**Keywords:** end of life, goals of care, outcome forecasting, rapid response systems, resource allocation

## Introduction

The conversation regarding allocation of scarce resources has grown into the forefront of hospital systems worldwide, in particular since the start of the COVID-19 pandemic (1, 2). Supply chain disruptions, staffing concerns, and equipment shortages have exposed vulnerabilities within the hospital resource system that have further increased scrutiny of clinician decisions regarding resource allocation (3–5). The landscape of delivering patient care in this environment has been wrought with ethical considerations and earnest, widespread efforts geared toward providing transparent, equitable, and efficient health care (6, 7). Additionally, numbers of bedside staff have left the medical field due to the moral injuries developed through the administration and escalation of care deemed futile and inconsistent with the best wishes of their patients (8–12). Thus, investing the right resources in the right patients is a major healthcare priority.

## Rapid response systems

A resurgent expansion in rapid response systems (RRS) across the healthcare field has also occurred since the COVID-19 pandemic (13–15). RRS are safety mechanisms devised of an afferent limb (deterioration monitoring and hospital-specific rapid response criteria) that triggers efferent limb response personnel (typically a critical care registered nurse) capable of providing resources, skills, and potential interventions at the bedside (16–18). The overarching goals of these systems are to intervene prior to a major acute life-threatening deterioration, increase patient safety, and reduce potentially inappropriate or avoidable escalation of resources such as intensive care unit (ICU) bed utilization (19–21). While complexity and variability between RRS themselves, as well as their methods of documentation of outcomes and standardization of alert triggers, have made their impact difficult to study on a larger scale, there is promise in their use as a means of reducing unexpected mortalities (22). Goals of care (GOC) discussions and palliative care consultations often occur shortly after RRS activation and escalation to the ICU, leading to transition to comfort measures almost immediately upon arrival to the ICU, with those who are escalated to ICU status often demonstrating higher degrees of short-term mortality (23–25).

## Discussion

The skill and expertise of more fully developed RRS provider teams enable the utilization of earlier, directed, emergent goals of care discussions to prevent inappropriate escalation of care and allocation of resources toward patients unlikely to derive benefit (e.g., unlikely to survive with acceptable quality of life) (26–28). The role of the intensivist practitioner staffing RRS teams in end-of-life discussions outside of the ICU, and preceding escalation, is a growing concept that seeks to intervene earlier in patient care courses to prevent potentially untoward and unjust patient outcomes (29). These teams are also equipped with the skills and experience to assist primary teams in providing end-of-life care outside of the ICU, including in conjunction with specialized palliative care teams (30). Intensivists and RRS clinicians are also experienced in leading decision making around patient understanding and goals of care expectations, including desirable, acceptable, and realistic functional status following their clinical course (31).

In addition to the RRS team members' robust experience in caring for the critically ill, the stronger focus and broader involvement of these teams allow for an opportunity to also focus on the development of high-quality communication skills in clinician teams outside of palliative care consultant services. Primary team communication with their patients may be limited by lack of time, structured education, and experience with acutely ill patients at the end of life (32, 33). RRS team members are uniquely granted dedicated, individualized time with these acutely ill patients where they can utilize previous structured experience and may provide a fresh perspective to deteriorating patients and their families beyond what primary team clinicians can provide. These RRS teams also present a prime opportunity for simulated educational interventions aimed at improving, incentivizing, and utilizing high-quality communication skills during end-of-life discussions with patients, their providers, and healthcare proxies (34).

## Future directions

As life expectancy in the United States and abroad continues to increase, along with projected increases in potential disability free-living, high-fidelity goals of care discussions led by experienced providers utilizing accurate outcome forecasting become paramount to delivering patient centric care (35–37). Pairing clinician RRS team skillsets with predictive modeling to identify patients with low likelihood of acceptable quality of life survival could further reduce inappropriate allocation of resources and potential futile escalations of care, as well as provide more patient centered and data driven GOC discussions.

Two primary factors involved in identifying and predicting patient outcomes following clinical deterioration and subsequent RRS activation are baseline patient disease status (i.e., comorbid disease burden prior to admission) and the severity of the acute illness and deterioration. While studies have characterized patient morbidities (e.g., older age, diagnosis of frailty) potentially benefiting from more robust GOC discussions surrounding and preceding their acute clinical deterioration, there are currently no automated or dynamic tools validated to identify post-deterioration functional outcomes and high short-term mortality rates signaling possible futile care (38–41).

Furthermore, use of the electronic health record (EHR) capabilities continues to expand, with easily available data from the EHR holding valuable potential for providing a vehicle for clinicians to access live patient outcome predictions during important care decision points such as RRS activations (42). While EHR functionality has been explored extensively in predicting upcoming acute deterioration of patients, less has been done at incorporating this data into active machine learning models to provide primary team, RRS team, or ICU clinicians with data regarding potential mortality immediately following their hospitalization, as well as forecast the post-hospitalization disposition and perseverance of functional status following their potential deterioration (43, 44).

Future studies are warranted at incorporating current technological advancements to provide more timely, humane, patient centered, and data driven GOC discussions geared toward providing our patients with end-of-life care in line with their ultimate wishes. While this increase in utilization and integration of predictive machine learning systems into the clinical space holds great promise, it will do little to improve suboptimal communication between provider teams, families, and patients and warrants a multidisciplinary approach with an additional focus on improving communication skills of RRS team members and clinician providers. In addition to a broader approach, these technological integrations, while being data driven, serve as another entry point of provider bias and resource distribution inequities into the clinical space with potential medicolegal ramifications (45, 46). Careful implementation of these systems along with future inquiry regarding the possible impact the inherent bias of these algorithmic forecasting systems has on clinicians is warranted and crucial at maintaining delivery of patient centric humanistic care in these vulnerable patient populations.

Overall, the combination of RRS teams with timely EHR based data-driven goals of care conversations has the potential to help guide appropriate resource allocation and utilization to serve both patient and health system needs but requires careful consideration of communication skill development and implications of potential bias before broad implementation.

## References

[1] Delbon P Conti A Maghin F Falconi B. Resource scarcity and allocation criteria during the Covid-19 pandemic: ethical issues. J Public health Res. (2022) 11:22799036221106659. doi: 10.1177/22799036221106659

[2] Riccioni L Bertolini G Giannini A Vergano M Gristina G Livigni S . Clinical ethics recommendations for the allocation of intensive care treatments, in exceptional, resource-limited circumstances. Recenti Prog Med. (2020) 111:207–11. doi: 10.1701/3347.33183 32319442

[3] Koning ERD Schalij MJ Boogers MJ Beeres SL Kramer ID Dannenberg WJ. Managing hospital capacity: achievements and lessons from the COVID-19 pandemic. Prehosp Disaster Med. (2022) 37:600–8. doi: 10.1017/S1049023X22001169 35950299 PMC9411723

[4] Miller FA Young SB Dobrow M Shojania KG. Vulnerability of the medical product supply chain: the wake-up call of COVID-19. BMJ Qual Saf. (2021) 30:331–5. doi: 10.1136/bmjqs-2020-012133 33139342

[5] McMahon DE Peters GA Ivers LC Freeman EE Samy AM. Global resource shortages during COVID-19: bad news for low-income countries. PLoS Negl Trop Dis. (2020) 14:e0008412. doi: 10.1371/journal.pntd.0008412 32628664 PMC7337278

[6] Yuk-Chiu Yip J. Healthcare resource allocation in the COVID-19 pandemic: ethical considerations from the perspective of distributive justice within public health. Public Health Pract. (2021) 2:100111. doi: 10.1016/j.puhip.2021.100111 33817679 PMC8005252

[7] Rawlings A Brandt L Ferreres A Asbun H Shadduck P. Ethical considerations for allocation of scarce resources and alterations in surgical care during a pandemic. Surg Endosc. (2021) 35:2217–22. doi: 10.1007/s00464-020-07629-x 32399942 PMC7216853

[8] Ho MH Lin CC. Futile care and burnout in intensive care unit nurses. Intensive Crit Care Nurs. (2022) 71:103228. doi: 10.1016/j.iccn.2022.103228 35397979

[9] Schouela N Kyeremanteng K Thompson LH Neilipovitz D Shamy M D'Egidio G. Cost of futile ICU care in one Ontario hospital. Inquiry (2021) 58:469580211028577. doi: 10.1177/00469580211028577 34218711 PMC8261843

[10] Ford EW. Stress, burnout, and moral injury: the state of the healthcare workforce. J Healthc Manag. (2019) 64:125–7. doi: 10.1097/JHM-D-19-00058 31999259

[11] Guttormson JL Calkins K McAndrew N Fitzgerald J Losurdo H Loonsfoot D. Critical care nurse burnout, moral distress, and mental health during the COVID-19 pandemic: a United States survey. Heart Lung (2022) 55:127–33. doi: 10.1016/j.hrtlng.2022.04.015 35561589 PMC9050623

[12] Mantri S Song YK Lawson JM Berger EJ Koenig HG. Moral injury and burnout in health care professionals during the COVID-19 pandemic. J Nerv Ment Dis. (2021) 209:720–6. doi: 10.1097/NMD.0000000000001367 34582400

[13] Mitchell OJL Doran O Yuriditsky E Root C Teran F Ma K . Rapid response system adaptations at 40 US hospitals during the COVID-19 pandemic. Resusc Plus (2021) 6:100121. doi: 10.1016/j.resplu.2021.100121 33870236 PMC8041183

[14] Rocha HAL Alcântara ACdC Netto FCdB Ibiapina FLP Lopes LA Rocha SGMO . Dealing with the impact of the COVID-19 pandemic on a rapid response team operation in Brazil: quality in practice. Int J Qual Health Care (2021) 33:mzaa114. doi: 10.1093/intqhc/mzaa114 32991710 PMC7543504

[15] Steel AC Reynolds SF. The growth of rapid response systems. Jt Comm J Qual Patient Saf . (2008) 34:489–95, 433. doi: 10.1016/S1553-7250(08)34062-8 18714752

[16] Jeddian A Lindenmeyer A Marshall T Howard A Sayadi L Rashidian A . Implementation of a critical care outreach service: a qualitative study. Int Nurs Rev. (2017) 64:353–62. doi: 10.1111/inr.12377 28555783

[17] Snarskis C Banerjee A Franklin A Weavind L. Systems of care delivery and optimization in the postoperative care wards. Anesthesiol Clin. (2023) 41:875–86. doi: 10.1016/j.anclin.2023.05.007 37838390

[18] Jones DA DeVita MA Bellomo R. Rapid-response teams. N Engl J Med. (2011) 365:139–46. doi: 10.1056/NEJMra0910926 21751906

[19] Maharaj R Raffaele I Wendon J. Rapid response systems: a systematic review and meta-analysis. Crit Care (2015) 19:254. doi: 10.1186/s13054-015-0973-y 26070457 PMC4489005

[20] Chan PS Jain R Nallmothu BK Berg RA Sasson C. Rapid response teams: a systematic review and meta-analysis. Arch Intern Med. (2010) 170:18–26. doi: 10.1001/archinternmed.2009.424 20065195

[21] Dukes K Bunch JL Chan PS Guetterman TC Lehrich JL Trumpower B . Assessment of rapid response teams at top-performing hospitals for in-hospital cardiac arrest. JAMA Intern Med. (2019) 179:1398–405. doi: 10.1001/jamainternmed.2019.2420 31355875 PMC6664378

[22] Piasecki RJ Himmelfarb CRD Gleason KT Justice RM Hunt EA. The associations between rapid response systems and their components with patient outcomes: a scoping review. Int J Nurs Stud Adv. (2023) 5:100134. doi: 10.1016/j.ijnsa.2023.100134 38125770 PMC10732356

[23] Jones D Moran J Winters B Welch J. The rapid response system and end-of-life care. Curr Opin Crit Care (2013) 19:616–23. doi: 10.1097/MCC.0b013e3283636be2 23799463

[24] Sulistio M Franco M Vo A Poon P William L. Hospital rapid response team and patients with life-limiting illness: a multicentre retrospective cohort study. Palliat Med. (2015) 29:302–9. doi: 10.1177/0269216314560802 25634630

[25] Friesen JN Wanberg EJ Allman A LeMahieu A Pennington K Gallo de Moraes A. Rapid response events prompt increased goals of care discussions and code status changes. Am J Hosp Palliat Med. (2025) 1–8. doi: 10.1177/10499091251363899 40729745

[26] Tam B Salib M Fox-Robichaud A. The effect of rapid response teams on end-of-life care: a retrospective chart review. Can Respir J. (2014) 21:302–6. doi: 10.1155/2014/393807 25299222 PMC4198233

[27] Zhukovsky DS Heung Y Enriquez P Itzep N Lu Z Nortje N . Just-in-time decision making: preliminary findings of a goals of care rapid response team. J Pain Symptom Manage. (2023) 65:e337–43. doi: 10.1016/j.jpainsymman.2022.11.022 36496112 PMC9729166

[28] Erath A Shipley K Walker LA Burrell E Weavind L. Code status at time of rapid response activation - impact on escalation of care? Resusc Plus. (2021) 6:100102. doi: 10.1016/j.resplu.2021.100102 34223364 PMC8244475

[29] Hilton AK Jones D Bellomo R. Clinical review: the role of the intensivist and the rapid response team in nosocomial end-of-life care. Crit Care (2013) 17:224. doi: 10.1186/cc11856 23672813 PMC3672544

[30] Nelson JE Mathews KS Weissman DE Brasel KJ Campbell M Curtis JR . Integration of palliative care in the context of rapid response: a report from the improving palliative care in the ICU advisory board. Chest (2015) 147:560–9. doi: 10.1378/chest.14-0993 25644909 PMC4314822

[31] Fried TR Bradley EH Towle VR Allore H. Understanding the treatment preferences of seriously ill patients. N Engl J Med. (2002) 346:1061–6. doi: 10.1056/NEJMsa012528 11932474

[32] White DB Andersen SK. Conversations on goals of care with hospitalized, seriously ill patients. JAMA (2023) 329:2021–2. doi: 10.1001/jama.2023.8970 37210664

[33] Bernacki RE Block SD American College of Physicians High Value Care Task Force. Communication about serious illness care goals: a review and synthesis of best practices. JAMA Intern Med. (2014) 174:1994–2003. doi: 10.1001/jamainternmed.2014.5271 25330167

[34] Curtis JR Lee RY Brumback LC Kross EK Downey L Torrence J . Improving communication about goals of care for hospitalized patients with serious illness: study protocol for two complementary randomized trials. Contemp Clin Trials (2022) 120:106879. doi: 10.1016/j.cct.2022.106879 35963531 PMC10042145

[35] Beltran-Sanchez H Soneji S Crimmins EM. Past, present, and future of healthy life expectancy. Cold Spring Harb Perspect Med. (2015) 5:a025957. doi: 10.1101/cshperspect.a025957 26525456 PMC4632858

[36] Devasia TP Howlader N Dewar RA Stevens JL Mittu K Mariotto AB. Increase in the life expectancy of patients with cancer in the United States. Cancer Epidemiol Biomarkers Prev. (2024) 33:196–205. doi: 10.1158/1055-9965.EPI-23-1006 38015774 PMC10872878

[37] Arias E Xu J Kochanek K. United States life tables, 2022. Natl Vital Stat Rep. (2025) 1:174575. doi: 10.15620/cdc/174575 40493958 PMC12359762

[38] Shappell C Snyder A Edelson DP Churpek MM Investigators AHAGWTG. Predictors of in-hospital mortality after rapid response team calls in a 274 hospital nationwide sample. Crit Care Med. (2018) 46:1041–8. doi: 10.1097/CCM.0000000000002926 29293147 PMC6044728

[39] Subramaniam A Tiruvoipati R Green C Srikanth V Soh L Yeoh AC . Frailty status, timely goals of care documentation and clinical outcomes in older hospitalised medical patients. Intern Med J. (2021) 51:2078–86. doi: 10.1111/imj.15032 32892457

[40] Dugan C Weightman S Palmer V Schulz L Aneman A. The impact of frailty and rapid response team activation on patients admitted to the intensive care unit: a case-control matched, observational, single-centre cohort study. Acta Anaesthesiol Scand. (2024) 68:794–802. doi: 10.1111/aas.14418 38576212

[41] Sharp D McKenzie D Padayachee L Subramaniam A. Frailty as a trigger for goals-of-care discussions in rapid response calls: a single-centre retrospective cohort study. Aust Crit Care (2025) 38:101090. doi: 10.1016/j.aucc.2024.06.011 39127605

[42] Esfahani S Yi C Madani CA Davidson JE Edmonds KP Wynn S. Exploiting technology to popularize goals-of-care conversations and advance care planning. Crit Care Nurse (2020) 40:32–41. doi: 10.4037/ccn2020576 32737487

[43] Steitz BD McCoy AB Reese TJ Liu S Weavind L Shipley K . Development and validation of a machine learning algorithm using clinical pages to predict imminent clinical deterioration. J Gen Intern Med. (2024) 39:27–35. doi: 10.1007/s11606-023-08349-3 37528252 PMC10817885

[44] Singh K Valley TS Tang S Li BY Kamran F Sjoding MW . Evaluating a widely implemented proprietary deterioration index model among hospitalized patients with COVID-19. Ann Am Thorac Soc. (2021) 18:1129–37. doi: 10.1513/AnnalsATS.202006-698OC 33357088 PMC8328366

[45] Hofweber T Walker RL. Machine learning in health care: ethical considerations tied to privacy, interpretability, and bias. N C Med J. (2024) 85:240–5. doi: 10.18043/001c.120562 39466090 PMC13368204

[46] Hanna MG Pantanowitz L Jackson B Palmer O Visweswaran S Pantanowitz J . Ethical and bias considerations in artificial intelligence/machine learning. Mod Pathol. (2025) 38:100686. doi: 10.1016/j.modpat.2024.100686 39694331

